# A Review and Case Discussion on a Rare Cause of Non-cirrhotic Portal Hypertension

**DOI:** 10.7759/cureus.30252

**Published:** 2022-10-13

**Authors:** Yu Bin Tan, Jonathan Guo Xiang Teh, Yee Yen Gwee, Yi Kang Ng

**Affiliations:** 1 Gastroenterology and Hepatology, Sengkang General Hospital, Singapore, SGP; 2 Pathology, Singapore General Hospital, Singapore, SGP

**Keywords:** varices, chemotherapy, xelox, oxaliplatin, nodular regenerative hyperplasia, non-cirrhotic portal hypertension

## Abstract

Nodular regenerative hyperplasia (NRH) is a rare cause of non-cirrhotic portal hypertension that should be considered in patients with no risk factors for chronic liver disease or in any unusual presentation of variceal hemorrhage. We present a case of an 82-year-old Chinese female, with a history of previous metastatic sigmoid carcinoma with oxaliplatin use, who presented with melena. A gastroscopy done revealed one column of grade 3 esophageal varix, two columns of grade 2 esophageal varices, and a type 1 gastroesophageal varix with stigmata of recent hemorrhage. Cyanoacrylate glue therapy was performed without any complications. A follow-up computed tomography (CT) imaging of the abdomen did not reveal any significant features of cirrhosis or venous thrombosis, and the decision was made for a transjugular liver biopsy with hepatic venous pressure gradient (HVPG) measurement. The measured HVPG was 6 mmHg, and the liver biopsy showed features consistent with NRH.

## Introduction

Portal hypertension is a syndrome characterized by the formation of portosystemic collaterals in the presence or absence of cirrhosis. A key feature of portal hypertension is the formation of varices commonly found in the esophageal and gastric regions and poses a risk of bleeding due to the inherent high pressures within them. The most common etiology of portal hypertension is liver cirrhosis, and gastric varices feature less commonly in cirrhotic portal hypertension, compared with esophageal varices, with an incidence rate of only 2%-20% [1].

Nodular regenerative hyperplasia (NRH) is a rare vascular disorder of the liver that forms part of the porto-sinusoidal vascular disorder (PSVD) spectrum and is one of the causes of non-cirrhotic portal hypertension. It was first described as “miliary hepatocellular adenomatosis” by Ranstrom in 1953 in a patient with Felty’s syndrome and commonly manifests as splenomegaly, thrombocytopenia, and esophageal varices [2-4]. A classification system proposed by Wanless et al. provided histological criteria for the diagnosis of nodular regenerative hyperplasia (NRH) [5]. The criteria include the presence of hepatocellular nodules less than 3 mm in diameter that were not surrounded by fibrosis.

## Case presentation

An 82-year-old Chinese female, with a significant history of previous sigmoid cancer in 2001 status post high anterior resection with four cycles of adjuvant oxaliplatin and capecitabine (XELOX) completed in 2005, was referred for melena. Although appearing pale, her abdomen was soft and non-tender, and she was anicteric without any stigmata of chronic liver disease. Laboratory investigations showed long-standing thrombocytopenia (100-120 × 10^9^/L) since 2005 with a previous computed tomography (CT) imaging of the abdomen in 2013 showing a smooth non-nodular liver with no parenchymal changes and no features of portal hypertension such as splenomegaly. She was on follow-up with hematology, although no clear etiology was found for the thrombocytopenia. Liver enzymes and coagulation profile were also normal. Nevertheless, she was started on esomeprazole infusion, somatostatin infusion, and ceftriaxone and was listed for a gastroscopy the very same day.

**Figure 1:**
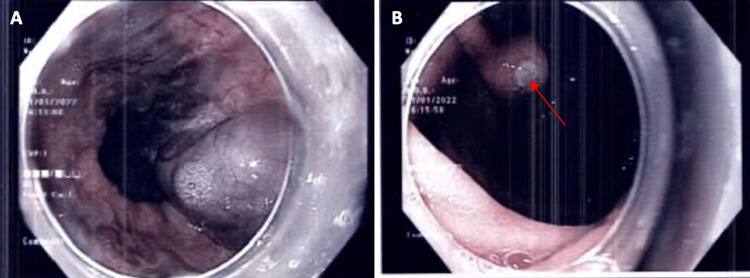
(A) Large column of esophageal varices seen on entry with no stigmata of recent hemorrhage. (B) Gastric varix with prominent platelet-fibrin plug (red arrow) seen in the gastric fundus.

On intubation of the esophagus, three columns of esophageal varices (one grade 3 and two grade 2) with no stigmata of recent hemorrhage were seen. However, on careful inspection of the fundus of the stomach, the large column varix seen in the esophagus extends over the cardia and the lesser curve (Sarin classification type 1 gastroesophageal varix). There was also a prominent platelet-fibrin plug on the surface of this gastric varix suggestive of recent bleeding as shown in Figure 1. A decision was made for cyanoacrylate glue therapy, which was successfully performed without any complications.

Subsequently, a CT scan of the abdomen was performed to evaluate the etiology of the underlying portal hypertension. The scan revealed no significant features of liver cirrhosis or splenomegaly, and the portal vein, superior mesenteric veins, and splenic and hepatic veins were all patent, although the right portal vein caliber was relatively small. No significant ascites or hepatic mass was visualized. Blood investigations, such as antinuclear antibodies, anti-smooth muscle antibodies, anti-liver antibodies, ceruloplasmin, iron studies, immunoglobulins, and hepatitis B and C serologies, sent off to evaluate for other causes of liver disease also returned negative. A decision was thus made to proceed with a transjugular liver biopsy with hepatic venous pressure gradient (HVPG) measurement.

**Figure 2:**
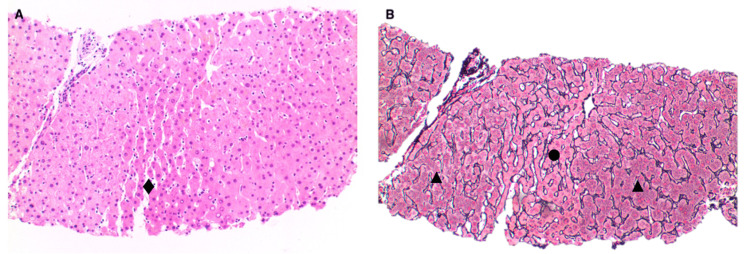
(A) The liver parenchyma is near normal with some dilated sinusoids (♦) on hematoxylin and eosin (H&E). (B) Reticulin shows alternating zones of hypertrophic (▲) and atrophic (●) hepatocytes.

A venogram was performed during the procedure, which showed multiple hepatic venous shunting of all three hepatic veins on balloon occlusion. The middle hepatic vein was used for measurement with the wedged pressure being 20 mmHg, free pressure being 14 mmHg, and HVPG being 6 mmHg. Liver biopsy samples obtained showed an adequate number of portal tracts and were of good quality. Examination of the liver core biopsy showed a relatively near-normal histological appearance, with patches of sinusoidal dilatation associated with atrophied hepatocytes. Features of nodular regenerative hyperplasia are better accentuated on reticulin stain, where linear zones of atrophic hepatocytes alternating with relatively hypertrophied hepatocytes are seen (Figure 2). Connective tissue stains are negative for any significant fibrosis such as bridging fibrosis and cirrhosis.

## Discussion

NRH is characterized by a diffused transformation of normal hepatic parenchyma into small regenerative nodules with little or no fibrosis. It is commonly associated with rheumatological, hematological, and congenital conditions, as well as medications, and while the pathogenesis is not clear, it is hypothesized to involve endothelial damage, leading to the obliteration of small portal veins throughout the liver, resulting in reduced blood flow in these areas and increase blood flow in other areas, leading to nodule formations via a hypertrophy response [5,6]. Oxaliplatin-based chemotherapy, such as XELOX, has been reported to have deleterious effects on sinusoidal endothelial cells, resulting in sinusoidal dilatation, congestion, and obstruction. These sinusoidal lesions result from the depletion of sinusoidal glutathione and the activation of matrix metalloproteinases and thus the overproduction of reactive oxygen species by oxaliplatin [7]. It has also been postulated that such sinusoidal lesions prevent liver regeneration and worsen portal pressures, leading to portal hypertension in 4.6% of such cases [5,8-10].

A systematic review was carried out on the electronic database PubMed® with the following keywords: non-cirrhotic portal hypertension, nodular regenerative hyperplasia, oxaliplatin, XELOX, chemotherapy, and varices. Further manual searches were conducted using references from related articles. The literature review was current as of August 2022. A total of 197 articles were reviewed, and 18 articles were found to be relevant. There are several case reports and a few cohort studies of patients with metastatic colorectal cancer treated with chemotherapy regimens that included oxaliplatin, who subsequently had liver resection or biopsy, and were found to have NRH. The incidence of NRH in these cohort studies ranges from as low as 0.5% to as high as 24.5% [11-16]. A retrospective cohort study of 24 patients with NRH showed that 83% of patients had splenomegaly, 75% had gastroesophageal varices, and 46% had ascites. All patients had liver function parameters that were normal or only slightly deranged, and the HVPG was lower than 10 mmHg in 71% of these patients, thus highlighting the pre-sinusoidal component of their portal hypertension [17]. Interestingly, a large cohort study conducted by Vigano et al. on 429 patients showed that NRH regresses after nine months off oxaliplatin and/or irinotecan-based chemotherapy regimens, with a prevalence of 6.5% versus 20.1% (p=0.063) [15].

Management of varices in non-cirrhotic portal hypertension would include endoscopic variceal ligation for esophageal varices and cyanoacrylate glue injection for gastric varices. Data is lacking to recommend the use of non-selective beta-blockers for both primary and secondary prophylaxis for these varices. Portosystemic shunting can be considered in cases of uncontrolled or recurrent portal hypertensive bleeds and failed endoscopic therapy [18].

What was unusual in our patient was that she presented with a variceal bleed without any other clinical, biochemical, or radiological features of portal hypertension, such as splenomegaly or ascites. In addition, her first presentation of NRH was almost 17 years after her last chemotherapy, which was unusual based on current evidence and studies. Although she had chronic thrombocytopenia since her chemotherapy in 2005, she had been followed up with hematology regularly, and both autoimmune and hematological causes for her thrombocytopenia have yielded negative results. One can only postulate that her thrombocytopenia might have been due to hypersplenism without any splenomegaly, which is extremely rare.

## Conclusions

NRH is a rare cause of non-cirrhotic portal hypertension and should always be suspected in a patient who presents atypically as in this case. This patient presented with a gastric varix bleed that is uncommon in a cirrhotic patient. In addition, she did not have any risk factors or evidence to suggest liver cirrhosis, and thus, further workup with a liver biopsy was warranted. High suspicion for non-cirrhotic portal hypertension etiologies should be entertained.
